# Surfactant-induced enhancement of droplet adhesion in superhydrophobic soybean (*Glycine max* L.) leaves

**DOI:** 10.3762/bjnano.8.234

**Published:** 2017-11-08

**Authors:** Oliver Hagedorn, Ingo Fleute-Schlachter, Hans Georg Mainx, Viktoria Zeisler-Diehl, Kerstin Koch

**Affiliations:** 1 Faculty of Life Sciences, Rhine-Waal University of Applied Science, Marie-Curie-Straße 1, 47533 Kleve, Germany; 2 BASF Personal Care and Nutrition GmbH, Henkelstr. 67, 40589 Düsseldorf, Germany; 3 Department of Ecophysiology, IZMB, University of Bonn, Kirschallee 1, 53115 Bonn, Germany

**Keywords:** droplet adhesion, epicuticular wax, *Glycine max* L, superhydrophobic, surfactants

## Abstract

This study performed with soybean (*Glycine max* L.), one of the most important crops for human and animal nutrition, demonstrates that changes in the leaf surface structure can increase the adhesion of applied droplets, even on superhydrophobic leaves, to reduce undesirable soil contamination by roll-off of agrochemical formulations from the plant surfaces. The wettability and morphology of soybean (*Glycine max* L.) leaf surfaces before and after treatment with six different surfactants (Agnique^®^ SBO10 and five variations of nonionic surfactants) have been investigated. The leaf surface structures show a hierarchical organization, built up by convex epidermal cells (microstructure) and superimposed epicuticular platelet-shaped wax crystals (micro- to nanostructure). Chemical analysis of the epicuticular wax showed that 1-triacontanol (C_30_H_61_OH) is the main wax component of the soybean leaf surfaces. A water contact angle (CA) of 162.4° (σ = 3.6°) and tilting angle (TA) of 20.9° (σ = 10.0°) were found. Adherence of pure water droplets on the superhydrophobic leaves is supported by the hydrophilic hairs on the leaves. Agnique^®^ SBO10 and the nonionic surfactant XP ED 75 increased the droplet adhesion and caused an increase of the TA from 20.9° to 85° and 90°, respectively. Scanning electron microscopy showed that surfactants with a hydrophilic–lipophilic balance value below 10 caused a size reduction of the epicuticular wax structures and a change from Cassie–Baxter wetting to an intermediate wetting regime with an increase of droplet adhesion.

## Introduction

The cuticle, as the outermost layer of higher plant surfaces, represents the interface between plants and their environment and accomplishes essential functions to ensure the maintenance of a terrestrial plant life, such as the reduction of water loss [1], control of gas exchange [2–3], protection from harmful UV radiation [4] and aiding mechanical stability [5]. Furthermore, the cuticle interacts with its biotic environment and plays a crucial role for insect signaling [6] and insect attachment [7–9]. The leaf surfaces are composed of epidermis cells covered by a cuticle, which is a continuous extracellular membrane on primary plant tissues (shoots, leaves, fruits) of higher plants [10–11]. It is built up by a network of the cross-linked ester-like biopolymer, cutin, with integrated (intracuticular) and superimposed (epicuticular) waxes [12–13]. A large diversity of epicuticular wax chemistry and morphology has been described [14]. Epicuticular wax can either appear as a flat film covering the cuticle, or as a three-dimensional wax structure of various shapes having crystalline structure [15–16]. Cuticular wax, as defined in biology, represents a mixture of long-chain aliphatic hydrocarbons, such as alkanes, aldehydes, primary alcohols, fatty acids, ketones and esters [17–19]. In addition, aromatic compounds such as flavonoids and pentacyclic triterpenes can also been found. The chemical composition is genetically determined [20], and it shows considerable differences in different species [18] and can change on different organs of a plant [14]. Variations were also found between intra- and epicuticular waxes [21–22]. However the hydrophobic characteristic of epicuticular waxes and their three-dimensional micro- to nanometer-scaled structures play a crucial role in surface wettability.

The liquid interaction at solid–gaseous interfaces, usually termed wettability, can be measured as a static or dynamic contact angle [23]. Surfaces on which a water droplet forms a spherical shape are characterized by a high contact angle (CA). Plant surfaces with high contact angles above 150° are termed superhydrophobic [24–25]. Wettable surfaces, on which an applied drop of water tends to spread, have a low or zero-degree contact angle.

Whether droplets adhere on a surface or not is described by the contact angle hysteresis (CAH). It is measured as the difference between the advancing and receding angles of a droplet (CAH = CA_adv_ − CA_rec_). Hysteresis can also be characterized by measuring the tilt angle (TA) of a surface at which an applied droplet of water starts to move. If the droplet rolls with little resistance at a low inclination angle of the surface, the contact angle hysteresis is small.

The contact area of the solid surface and the applied liquid also depends on the wetting mode. In the Wenzel mode [26] an applied water droplet penetrates into cavities formed by the surface structures, increasing the contact area, and resulting in high hysteresis of the applied liquid. In Cassie–Baxter mode [27] air remains in the surface cavities below the liquid droplet, and the droplet sits partially on air [28]. On such surfaces, the adhesion of liquid is usually limited because the low hysteresis droplets roll off at low inclination angles of the surface.

Plant surfaces covered with hydrophobic wax crystals of a few hundreds of nanometers are usually water repellent. Many economically important crops like wheat (*Triticum aestivum*) or rice (*Oryza sativa*) are characterized by leaf surfaces with superhydrophobic properties, thus the uptake of water-based agrochemicals, e.g., herbicides, is low [29] because applied droplets are likely to roll off. Droplet adhesion can be improved by adding adjuvants, such as surfactants. Surfactants enhance the leaf surface wettability by reduction of the surface tension of applied liquids. Surfactants are amphiphilic molecules with a nonpolar, hydrophobic structural group together with a hydrophilic group [30]. Surfactants are characterized by their hydrophilic/lipophilic balance (HLB). In general, if they have a high HLB (>10) they enhance the penetration of herbicides with high-water solubility, while lipophilic surfactants with a low HLB (<10) effectively enhance the uptake of poorly water-soluble herbicides [31]. Numerical values of HLB range from zero to twenty.

For a few species and adjuvants, a dissolution or structural alteration of epicuticular wax has been described [32–34]. Here we studied the influence of six surfactants with different HLB values on the epicuticular wax structures and leaf wettability in order to identify those which are able to improve the droplet adhesion on tilted leaves. Soybean (*Glycine max* L.) has been chosen because of its importance in animal and human food production. The global demand for soy and soy derivatives (vegetable oil and animal feed) is expected to increase to over 300 million tons per year by 2020 [35]. It is known that different wax structures are based on different chemical compositions with different solubility in different solvents [36]. Thus the solubility of the epicuticular waxes in chloroform was studied by using a time-dependent solubilization of the waxes and gas chromatography (GC–FID) and mass spectroscopy (GC–MS).

## Results

### Micromorphology of the leaf surface

**Figure 1:**
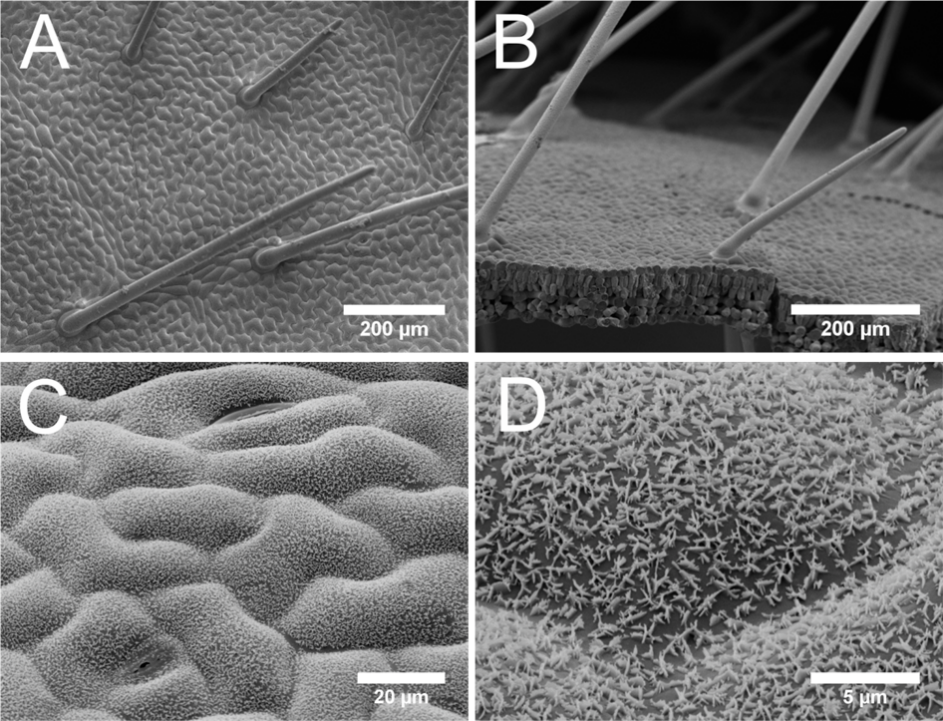
SEM micrographs of the untreated surface of a cryo-fixed sample of *Glycine max* L. leaves (upper leaf side) (A, B) and substituted with glycerol (C, D). In (A) a top view of the cryo-fixed adaxial surface is shown and the distribution of hairs and the undulated cell boundaries are illustrated. Figure 1B shows a side view of the leaf, showing a broken edge with hairs on the upper leaf side. In Figure 1C a detailed view of the slightly convex shape of the epidermal cells is shown. Figure 1D is a larger magnification image of Figure 1C, which shows the epicuticular wax platelets arranged in rosettes.

The micromorphology of leaf surfaces was investigated by scanning electron microscopy (SEM) and 3D light microscopy. Trichomes, commonly termed hairs, are slightly tilted, up to 2 mm in length, and are evenly distributed over the upper (adaxial) (Figure 1A,B) as well as the lower (abaxial) leaf sides. In contrast to the epidermal cells, the hairs were not covered with wax crystals. The epidermis cells are polygonal with undulated anticlinal cell boundaries (Figure 1C). The cells are slightly convex and densely covered with wax platelets, whereby clusters of five to ten platelets are situated with their narrow side perpendicular on the surface and are radially assembled in rosettes, which is the typical wax orientation feature of the legume family (Figure 1D). These wax platelets show fissured edges and, according to Barthlott et al. [14], are therefore called nonentire platelets. The heights of the wax platelets on both leaf sides varied between 1 to 1.2 µm because of their unregularly shape.

**Figure 2:**
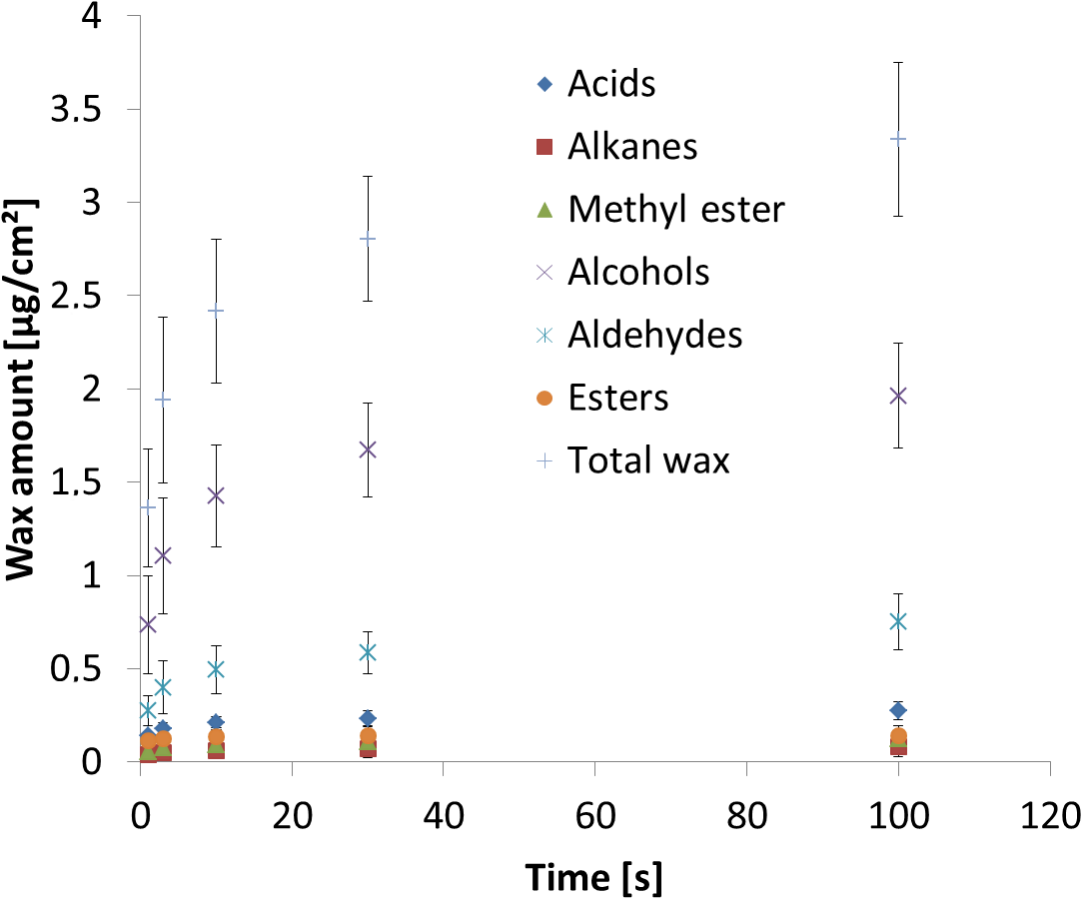
Extraction kinetics of soybean leaf wax by repetition of chloroform dipping. Absolute amounts [µg/cm²] extracted are plotted versus the total dipping times (1, 3, 10, 30, and 100 s) and extracted masses. Fraction 3 (10 s) represents the wax composition of a leaf which was dipped first for 1 s (fraction 1), than again for 2 s (fraction 2), and again for 7 s (fraction 3) in CHCl_3_. Each fraction was analyzed separately. Data points represent the mean (*n* = 4) ± standard deviation.

The wax isolation kinetics, tested by repeatedly dipping four leaves for different times into chloroform (Figure 2), demonstrates the solubility of the epicuticular waxes. The data show that a total wax extraction time of 10 s dissolves most of the epicuticular wax.

It can be seen that the highest amount of wax (1.4 ± 0.3 µg/cm^2^) was extracted after 1 s of dipping time (Figure 2). The amount of total extracted wax slightly increases for the following extraction steps, reaching a plateau (2.8 ± 0.36 µg/cm^2^) at 30 s. The solubility of the compound classes of fatty acids, alkyl esters, methylated alkyl esters and alkanes is constant with a dipping time of 10 s. Only for the aldehydes and primary alcohols was an increase in the solubility amount found when the leaves were dipped for 30 or 100 s. This increase for the aldehydes and primary alcohols at longer dipping times can be attributed to dissolved intracuticular wax, thus the 10 s dipping time in chloroform was used for the following wax analysis.

### Chemical wax composition

**Figure 3:**
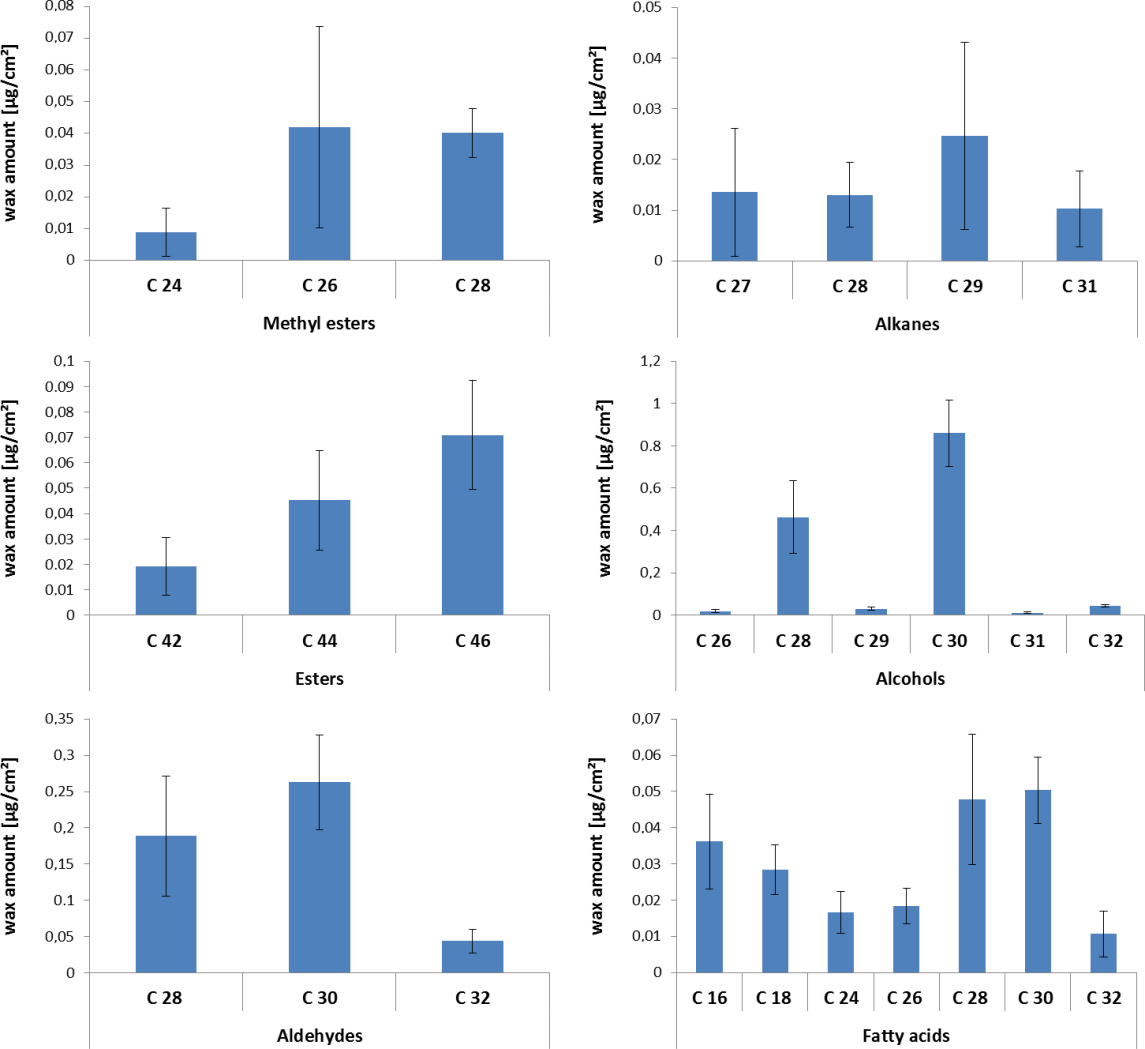
Chemical composition of soybean leaf wax divided by substance class. Wax was extracted by dipping the leaves for 10 s in pure chloroform. Bars represent the mean (*n* = 4) ± standard deviation.

The chemical composition of the extracted waxes analyzed by dipping individual leaves for 10 s in chloroform is shown in Figure 3. 26 individual wax components belonging to six major substance classes were identified. Primary alcohols ranging from C26 to C32 were the predominating compound class (1.42 ± 0.27 µg/cm^2^), which accounted for 58% of the total wax mass extracted. Triacontan-1-ol (0.86 ± 0.15 µg/cm^2^) and octacosane-1-ol (0.46 ± 0.17 µg/cm^2^) constitute the largest proportion. Further (0.50 ± 0.13 µg/cm^2^) aldehydes (C28–C32), (0.21 ± 0.03 µg/cm^2^) primary fatty acids (C16–C32), 0.14 ± 0.06 µg/cm^2^ alkyl esters (C42–C46), (0.09 ± 0.05 µg/cm^2^) methylated alkyl esters (C24–C28) and (0.06 ± 0.04 µg/cm^2^) alkanes (C27–C31), were found.

### Wettability

The wettability of *Glycine max* L. leaf surfaces was characterized by measuring the static contact angle and tilt angle before and after treatment with the different surfactants and as a control only with water. The mean static contact angle (CA) of 10 µL water droplets on only water-treated samples was 162.4° (σ = 3.6°) with a tilt angle (TA) of 20.9° (σ = 10.0°).

**Figure 4:**
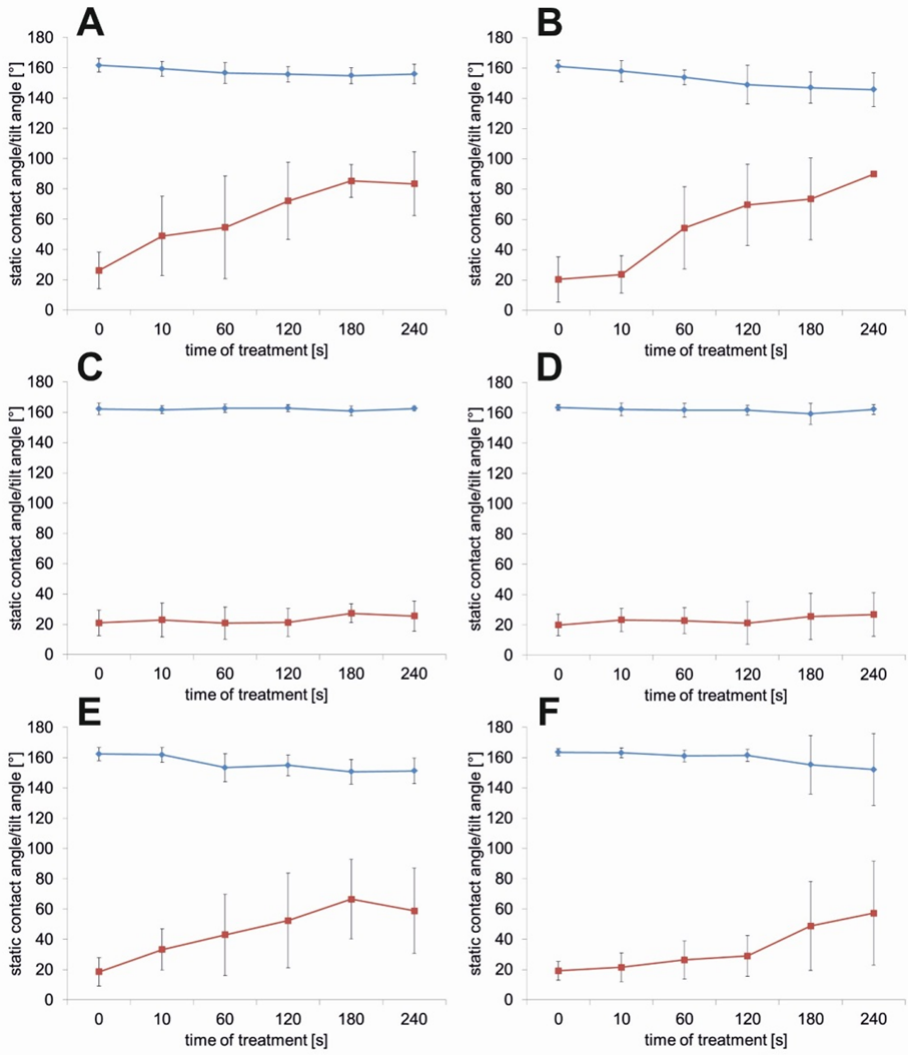
Contact angles (blue lines) and tilting angles (red lines) of the leaf surfaces of *Glycine max* L. after applying 20 µL of an aqueous surfactant solution (1 g/L) of SBO 10 (A), XP ED 00-75 (B), XP ED 28-11 (C), XP ED 28-12 (D), XP ED 28-13 (E), and XP ED 28-14 (F). Surfactants and leaf surfaces where in contact for 10, 60, 120, 180 and 240 s. Before the CAs and TAs were measured, the leaves were rinsed with pure water. The time zero represents the values for leaves which were in contact with water as a control. Values are given as the mean value of *n* = 10 ± standard deviation.

Surface treatment with the four nonionic surfactants, SBO 10, XP ED 00-75, XP ED 28-13 and XP ED 28-14, led to a slight decrease of the CA from 161° to 156°, 146°, 151° and 152°, respectively (Figure 4A,B,E,F), after a 240 s contact time, whereas the treatments with the surfactants XP ED 28-11 and XP ED 28-12 showed no distinct changes (Figure 4C,D). However the treatment with SBO 10 and XP ED 28-75 led to the fastest increase in the TA with a maximum of 83° and 90°, respectively, after 240 s. Additionally, XP ED 28-13 and XP ED 28-14 showed a significant increase to about 60°, whereas the treatment with XP ED 28-11 and XP ED 28-12 showed no distinct changes. A rapid and distinct increase in the TA was only found after treatment with SBO 10 and XP ED 28-13. Both surfactants increased the TA within 10 s of contact time for SBO 10 from a TA of 20° to a TA of 50°, and for ED 28-13 the TA increased from 16° to 30°.

### Structural and wetting changes caused by surfactants

**Figure 5:**
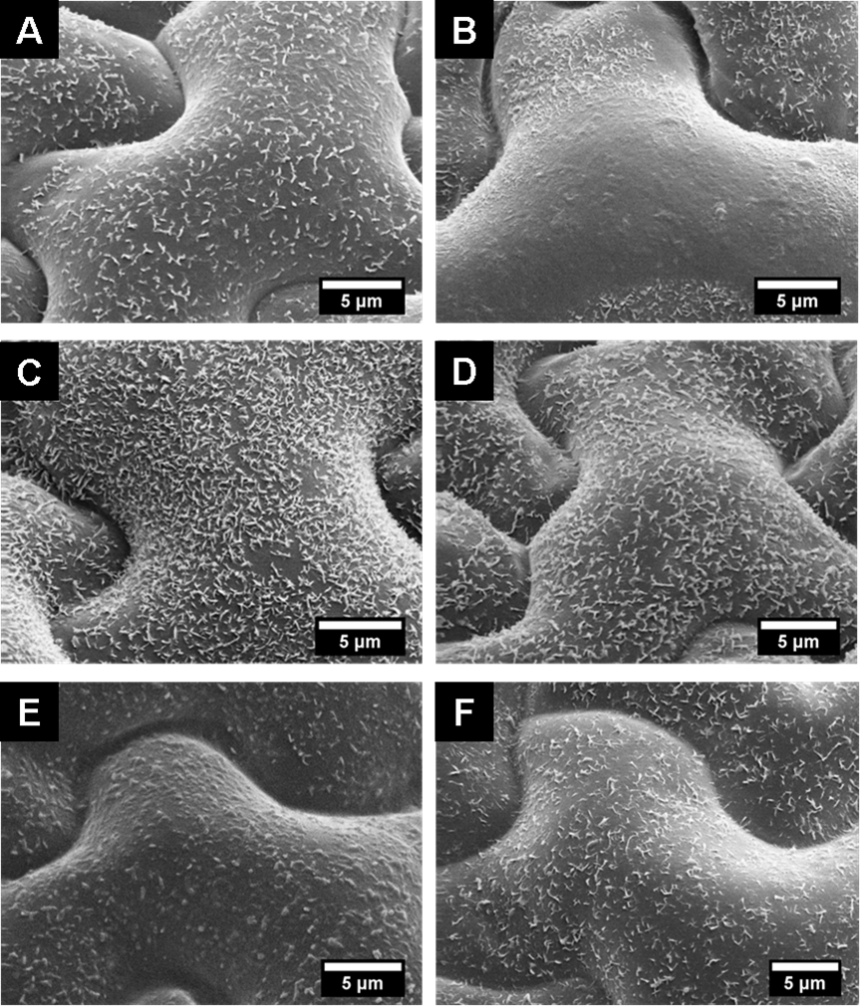
SEM micrographs of cryo-fixed upper leaf surfaces of *Glycine max* L. after 240 s of treatment with surfactants: SBO 10 (A), XP ED 00-75 (B), XP ED 28-11 (C), XP ED 28-12 (D), XP ED 28-13 (E), and XP ED 28-14 (F). For comparison of the wax structures see the SEM images before treatment in Figure 1D.

SEM images of the epicuticular wax crystals (EWCs) after treatment of leaf surfaces for 240 s with different surfactants showed alterations in different extensions, whereas the EWCs of water-treated leaves showed no structural changes. Alterations in the EWC structure after contact with the surfactants were caused by dissolution of the EWCs as shown in Figure 5. SBO 10 and XP ED 28-14 showed a reduction in density and height of the EWC from approximately 1–1.2 µm to below 0.5 µm (Figure 5A,F) compared to the water-treated leaf surfaces (Figure 1D). The most prominent changes in the EWC structure were caused by XP ED 00-75 (Figure 5B) where the wax crystals were dissolved in the exposed central areas of the epidermal cells. In the sunken part of the cell boundaries (anticlinal cell areas), wax crystals were partially dissolved so that only the bases of the wax crystals remained in these areas (Figure 5B). Also, after contact with XP ED 28-13 (Figure 5E), the wax platelets were dissolved but residue of small, granule-shaped wax structures were still present on the cells. Within the contact areas of XP ED 28-11 (Figure 5C) and XP ED 28-12 solutions (Figure 5D) the lowest structural changes of the EWC were found. In comparison to the untreated leaves, less densely covered cell areas were found.

**Figure 6:**
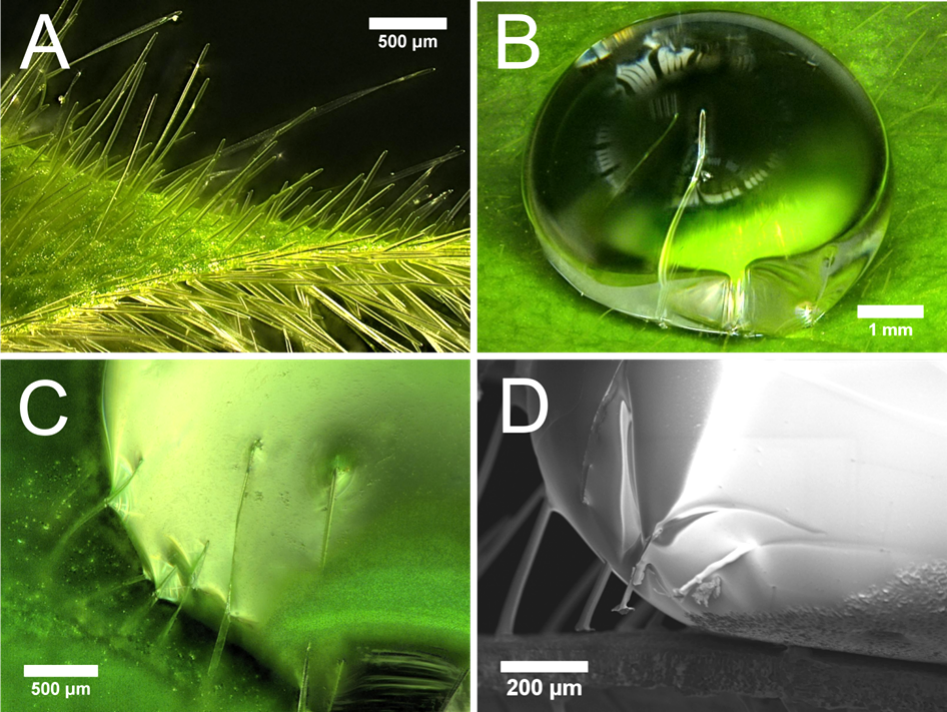
(A) 3D light microscopy images of fresh untreated leaf sample of *Glycine max* L. with long hairs on both leaf sides. The hydrophilic character of the hairs is demonstrated by penetration of the hairs into the droplet (B) and the concave meniscus which occurs when hairs come into contact with droplets resting on the surface (C). In (D) a scanning electron micrograph of a frozen glycerol–water droplet in contact with the leaf surface of *Glycine max* L. is shown.

Further 3D light microscopy studies revealed that the hairs penetrate into water droplets resting on the surface (Figure 6A,B) and prevent them from rolling off at lower inclination angles. The hydrophilic character of the hairs is demonstrated by the formation of a concave meniscus when a droplet comes into contact with the hairs (Figure 6C). By using the cryo-technique, frozen specimens could be investigated in an SEM and the interface between droplets containing a glycerol–water mixture and the epidermal cell surface could be investigated. The side view on the basis of an applied droplet in contact with the leaf surface is shown in Figure 6D. The cryo-technique allows mechanical manipulation of the frozen specimen so that parts of the leaf can be removed mechanically and the droplet contact area becomes visible. The SEM figure shows (see the right bottom area of the droplet) imprints within the droplet surface caused by the epicuticular wax, indicating that the droplet in a Cassie–Baxter state on the epicuticular wax layer.

## Discussion

### Chemical analysis

The isolation kinetics has been performed to identify the appropriate dipping time and to exclude extraction of intracuticular wax components in the subsequent performed wax analysis. The chemical analysis revealed that the soybean wax almost exclusively contains molecules with chain length greater than C_22_, primary aliphatic alcohols, but predominantly triacosanol. In previous work by Elmore et al. [37] the epicuticular wax of a different soybean variety has been qualitatively analyzed. A comparison of both analyses shows only small differences in the wax chemical composition of both varieties, but the described esters were missing in the work of Elmore et al. and also a lower amount of acid was found in the present study. However, such small differences might be caused by the different extraction times used. A recently published chemical analysis of soybean leaves (variety not defined) by Damato et al. [38] is based on a 30 s dipping time of the leaves in chloroform. The authors analyzed only “the strongest retention peaks” of the chromatogram, gave no quantitative data, and they only identified alkanes which represent the lowest amount of components in our analysis, and two compounds, the 9 octadecenamide (C_18_H_35_NO) and a diisooctyl adipate (C_22_H_42_O_4_) are not described for these waxes. However, none of the major compounds of primary alcohols, aldehydes, esters or acids found by Elmore et al. [37] and those presented here in this analysis are described by Damato et al. [38].

### Wettability

Contact angle measurements of pure water revealed the superhydrophobic properties of *Glycine max* L. leaves (CA 162.4° ± 3.6°). The superhydrophobic properties of soybean leaves are established by its surface sculptures built up by convex polygonal cells and superimposed epicuticular wax crystals with its hydrophobic surface chemistry. Due to the formation of a secondary structure on top of the convex epidermal cells, the interfacial contact area between the water droplet and the leaf surface is notably reduced, thus usually leading to extreme water repellency [39–40]. In contrast to other superhydrophobic plant surfaces like the lotus leaf (*Nelumbo nucifera*), the leaf surface of *Glycine max* L. does not show self-cleaning properties, which are generally indicated by low tilting angle or low contact angle hysteresis. Cryo-SEM investigations showed that droplets of a water–glycerol mixture are in the Cassie–Baxter wetting regime with only partial contact to the plant surface. However, the hairs of the soybean leaves capture the applied water droplets at low inclination angles of the leaf (Figure 5). Since these hairs are not covered with wax crystals, they have a more hydrophilic character, which is indicated by the penetration of the hairs into the applied water droplets. This is in contrast to the wax-coated epidermal cells. Damato et al. [38] also measured the contact angle and hysteresis of water and water–surfactants on soybean leaves, but did not study the influence of the dense hydrophilic hair layer on droplet adhesion. The several millimeter long hairs are highly visible in the water droplets shown in the photographs captured with a goniometer. However, the authors believe that the wrinkling of the epidermis cells, which they found in the SEM investigation, was responsible for the large hysteresis of pure water-treated leaves. Wrinkling in the epidermis of well-prepared leaves for the SEM investigation was not observed in our investigations. Damato et al. [38] show SEM pictures with a strong wrinkling of the epidermis cells, presumably caused by water loss of the air-dried specimen.

Wetting on soybean leaves of different ages has recently been studied by Moran-Puente and Baur [41]. They found static contact angles for water of 149° (adaxial leaf side) with a trend showing a decrease of this value during leaf maturation. Large variations in the CA of water and various concentrations of water–surfactant mixtures were found within a single leaf and correlated to wax crystal density, whereby a less dense layer of wax crystals led to lower CAs. The fast dissolution of the epicuticular wax (here investigated over a time period of 4 min) correlates with an increase in the droplet adhesion, as shown by the increase of the tilt angle during the contact time. The treatment with nonionic surfactants of different HLB values showed a reduction in EWC density as well as in single crystal heights, caused by dissolution. Dissolution was most distinctive after treatment with the surfactants with a lower HLB value, which correlates with the hydrophilic and lipophilic character of the main wax compound triacontanol. XP E-D 00-75 and XP E-D 28-13 most effectively dissolved the wax crystals of the soybean leaves and thereby improved the adhesion of the applied water droplets. The crystal sizes and amount of wax crystals have a large influence on the wettability, significantly more so than the chemical composition of the waxes [42]. In vitro studies with wax crystals of different sizes and densities showed that superhydrophobicity requires wax crystal sizes of 400 nm in the case of wax tubules [42] and about 200 nm in the case of wax platelets composed of n-hexatriacontane [43]. Smaller sizes of wax crystals reduce the surface roughness and the contact angle and also increase the adherence of the droplets to the surfaces, as indicated by the hysteresis or given tilt angles [42–43]. Surfactants with a high HLB value (XP ED 28-11 and XP ED 28-12) and consequently more hydrophilic properties showed no measurable differences to water-treated samples. However, the surfactant XP ED 28-14 also has a high HLB value, but it showed a reduction in the density and height of the EWC correlated to an increase of the TA and a decreased CA. While XP ED 28-11 and XP ED 28-12 contain only diglycerol, XP ED 28-14 (a technical surfactant), is composed mainly of monoglycerol, with low amounts of tri- and tetraglycerol. Here it is assumed that the high amount of mono-glycerol, which is less polar, caused the wax alterations and therewith the better wettability. Based on this hypothesis, further investigation with pure glycerols with different chain lengths should be performed to compare the solubility of the waxes.

Early studies on plant surfaces of *Brassica oleracea var. gongylodes* L. [44] and *Beta vulgaris* L. [45] showed alterations of the EWC structure after treatment with Triton X-100. Surfactant residue in the peripheral area of the applied droplet (the “coffee drop effect”) described by Wolter [45] could not be observed. However they did not rinse the plant surfaces after treatment as was done here for CA measurements, thus the remaining wax residue was embedded in the surfactant and the degree of wax dissolution was difficult to determine. Nevertheless we cannot preclude that small amounts of surfactants (not visible in the SEM), residue on the leaves even after rinsing the surface with water.

### Dissolving of the wax crystals

According to Koch et al. [46–47] the main wax component determines the crystal shape of platelets and tubules. The wax crystal chemistry showed that the main component of *Glycine max* L. leaves consists of primary alcohols, especially triacontanol and octacosanol. Investigations on octacosan-1-ol crystal structure and scanning tunneling microscope (STM) analysis by Koch et al. [46] showed that polar groups of the primary alcohols are introverted and the nonpolar proportion is orientated to the outside of the platelet-shaped wax crystals. According to our results, it is concluded that surfactants with a lower HLB value led to a greater dissolution of the wax crystals built up by triacontanol by interacting with the nonpolar parts of the alcohol molecules. Most important for the increase in droplet adhesion is the above-discussed dissolution of wax crystals, which leads to a transition of the wetting regime from Cassie–Baxter mode to an intermediate wetting state or complete Wenzel wetting state, characterized by a high CA and a high TA. Differences in the solubility of wax types with different chemical composition have been described for organic solvents. Thus, plant species with different epicuticular wax chemical composition might also show differences in the alteration of their wax by surfactants.

## Conclusion

The presented data on the surface topography and chemistry of soybean leaf surfaces show that the hydrophobic compound of the leaves are three-dimensional epicuticular waxes, composed mainly of the primary alcohol triacosan-1-ol and octacosan-1-ol. An improvement of surface wetting and droplet adhesion was achieved by adding surfactants SBO 10, XP ED 0075 XP ED 28-13 with higher HLB values to the liquid. Such surfactants induce dissolution of the wax crystals and increase the droplet adhesion, as demonstrated by higher roll-off angles. Additionally, the droplet contact area was found to increase, as demonstrated by the decrease of the contact angle. However, the tilt angle of the soybean leaf after XP ED 28-14 treatment (having a high HLB) is not in accordance with our expectations and requires further investigation.

The results presented herein on the soybean surface microstructure, chemistry and wettability provide important information for the further development of more target specific formulations used for the improvement of foliar uptake of nutrients and for the increase in the efficacy in pest control by spray application techniques.

## Experimental

### Plant material

Plants of the black soybean *Glycine max* L. convar. max var. nigra-lutescens “Schwarze Poppelsdorfer” were cultivated in the trial fields of the Botanical Gardens of the University of Bonn (acquisition number BONN-19242). For SEM investigations and wax extraction, fully developed leaves at the second node at growth stage (GS) 13 of the BBCH decimal code (Biologische Bundesanstalt, Bundessortenamt und chemische Industrie) were harvested [48]. The leaves were transported and stored in a closable plastic box, equipped with a wet paper towel to prevent them from drying out before surface investigation.

### Wax extraction

The wax solubility in chloroform was characterized with an extraction kinetic to determine the dipping time when the epicuticular (but not intracuticular) wax was dissolved for chemical analysis. The extraction kinetic was obtained with four leaves harvested from different plants. Each leaf was successively dipped into five analytical vials containing 10 mL of chloroform (p.a. 99.8%, Merck, Darmstadt Germany) for 1 s (fraction 1), 2 s (fraction 2), 7 s (fraction 3), 20 s (fraction 4) and 70 s (fraction 5). Thus the total time for wax extraction was 100 s in fraction 5. The amount of wax extracted at the different dipping times was summed up for every single leaf prior to calculation of the mean and standard deviation. For the calculation of the wax extraction per leaf area, the surface area of the leaves was measured with a scanner and determined using standard software (Adobe Photoshop 6.0).

### Chemical wax analysis

The wax of four leaves, harvested from four different plants, was used for the chemical wax analysis. The epicuticular wax was extracted by dipping the leaves each into 10 mL of chloroform for 10 s at ambient temperature. The data presented in Figure 2 are mean values of four different chemical analyses. For quantification of wax molecules, *n*-tetracosane (10 µg, 99.5%, Fluka Chemika GmbH, Germany) was directly added to each of the samples as an internal standard. The chloroform volume of each sample was reduced under a gentle nitrogen stream at 60 °C to an end volume of 200 µL. The functional groups of acids and alcohols present in the plant wax were transformed to their corresponding trimethilsilyl esters and ethers by derivatization using 20 µL BSTFA (bis-*N*,*N*-(trimethylsilyl)trifluoroacetamide; Machery-Nagel GmbH & Co KG, Germany) and 20 µL of pyridine (Fluka, Germany) for 45 min at 70 °C in a heating block. The quantitative wax composition was analyzed by capillary gas chromatography (GC) (GC–FID; CG-Hewlett Packard 5890 series H, Hewlett-Packard, Palo Alto, CA, USA) equipped with a flame ionization detector. 1 µL of each sample were injected into the column (30 m DB-1 i.d. 0.32 mm, film 0.2 µm; J&W Scientific, Folsom, CA, USA), and H_2_ served as the carrier gas. For quantification of single components, peak areas were estimated using the software Enhanced ChemStation (G 1701DA Version D.00.00.38, Agilent Technologies 1989-2001). The identification of wax compounds was achieved by analyzing 1 µL of sample by gas chromatography coupled to a mass spectrometer (GC–MS; quadrupole mass selective detector HP 5971, Hewlett-Packard, Palo Alto, CA, USA). Based on the characteristic fragmentation patterns, the wax components could be identified. In both quantitative and qualitative analyses, the following temperature program was used. The oven was heated to 50 °C when samples (1 µL) were injected into the column. The temperature was held at 50 °C for 2 min, increased at 40 °C min^−1^ to 200 °C, held for 2 min, increased again at 3 °C min^−1^ to 310 °C, and finally held at this temperature for 30 min.

### Surfactants and their application

Six nonionic surfactants provided by Cognis GmbH (BASF, Germany) with different hydrophilic and lipophilic ratios (hydrophilic–lipophilic balance, HLB) were used to investigate the influence on leaf epicuticular wax and wettability (Table 1). Agnique^®^ SBO10 is a commercially available surfactant (BASF), whereas all other tested surfactants are single components of SBO 10, which are noncommercial and therefore labeled EP (experimental product). The leaves of *Glycine max* L. were cut and fixed on a glass slide with double-sided adhesive tape (TESA, Beiersdorf, Germany). On the leaf samples, 20 µL of the aqueous surfactant solutions (at commonly used concentrations for ethoxylated seed oil adjuvants of 1 g/L [49–50]) were applied via a dispenser. The contact time between the leaves and the applied aqueous surfactant solutions was 10 s, 60 s 120 s, 180 s, and 240 s. The surfactant action was interrupted by rinsing the vertically positioned leaf samples with 10 mL of distilled water. Leaves on which 20 µL of distilled water were applied for 240 s were used as a control. The references were also rinsed with water to identify potential structural changes of the wax by the rinsing process. After surface drying, the static CA and TA within the surfactant-treated areas were measured. Subsequently, two samples of each treatment were freeze-dried in liquid nitrogen and investigated by SEM. The CA and TA measurements were performed ten times for each surfactant sample and reference sample after five different contact times. Limited by the leaf areas, a maximum number of five measurements could be performed on one leaf, thus approximately 700 leaves were used.

**Table 1:** Surfactants used for leaf surface treatment.

Name	Chemical description	HLB

Agnique SBO 10	triglycerol, ethoxylated	10
XP ED 00-75	triglycerol, ethoxylated, acetylated	9.7
XP ED 28-11	diglycerol, ethoxylated, monoester	13.4
XP ED 28-12	diglycerol, ethoxylated, diester	10.7
XP ED 28-13	diglycerol, ethoxylated, tetraester	7.3
XP ED 28-14	diglycerol, ethoxylated	13.4

### Contact angle measurements

For the contact angle measurements (before and after application of the surfactant solutions), leaf samples of approximately 1 cm^2^ were cut from the central area of the leaves and were fixed onto microscope glass slides with double-sided adhesive tape. Each value was obtained after applying a 10 µL droplet of distilled water to the surface. The static contact angle was measured using an OCA 35 goniometer (Dataphysics, Filderstadt, Germany). The droplet shape was recorded with a horizontal CCD camera and was automatically calculated using the Laplace–Young fitting algorithm in the integrated software (SCA 20). Subsequently, the tilt angle was measured using a tilting device (TBU 30). The mean and median values were calculated from *n* = 10 measurements.

### Scanning electron microscopy (SEM)

Investigations of the leaf morphology by SEM were either performed with a Cambridge Stereoscan 200 (Cambridge Instruments GmbH, Dortmund) instrument or with a high-resolution field emission scanning electron microscope (FE-SEM, Gemini Supra 40VP, Carl Zeiss GmbH, Oberkochen, Germany), equipped with a cryo-preparation unit (Emitech K1250X, Gabler Instrumente GmbH, Germany), consisting of a freezing chamber, preparation chamber with integrated micromanipulator, sublimation unit and sputter coater. Cryo-preparation and glycerol substitution were used to avoid alterations in cell shape wax structure. Glycerol substitution ensures high electrical conductivity of the specimen and minimizes distortion from desiccation, without changing the leaf surface structures [36,51]. The leaves were cut into 0.5 cm^2^ pieces and placed on a wet paper towel inside a petri dish. Glycerol (90%) was added drop-wise over a period of 20 h. The process ensures that glycerol infiltrates the tissue through the cut edges without wetting the top face, which could potentially alter it. For SEM examinations, the cut edges were sealed with conductive carbon cement (Leit-C, Plano GmbH, Germany) and samples were optionally sputter-coated.

### Cryo-preparation for SEM

Areas of approximately 1 cm^2^ were cut out of the fresh leaf samples, fixed on a metal sample holder with a 1:1 mixture of conductive carbon cement (Leit-C, Plano GmbH, Germany) and glue (Tissue Tec O.C.T., Sakura Finetec Europe B.V., Zoeterwoude, Netherlands) and subsequently immersed into liquid nitrogen. The frozen leaves were fractured using a micromanipulator. Prior to cryo-fixation, the liquid nitrogen was cooled down in a separate freezing chamber at 10^−2^ mbar to −210 °C in order to reduce the air layer between liquid nitrogen and the sample. After cryo-fixation, the samples were permanently stored in a precooled (−140 °C) and evacuated (5 × 10^−4^ mbar) atmosphere to avoid ice crystal formation on the plant surfaces. The samples were platinum-coated before transferring them into the precooled (−140 °C) SEM chamber. The transfer of the frozen samples was performed using an evacuated (10^−2^ mbar) transfer chamber. The cryo-fixed samples were examined with acceleration voltages of 1 kV to 5 kV and working distances between 3 mm and 6 mm. The height of the wax platelets was measured at a 90° tilt angle of the specimen in order to get a side view of the crystals in the central field of the cells.

### Three-dimensional digital light microscopy

To visualize the topography of the leaves, a 3D light microscope (Keyence VHX-600DSO, Keyence Deutschland GmbH, Germany) was used. *Glycine max* L. leaves were fixed on a flat glass slide with double-sided adhesive tape (Tesa, Germany) and images were taken from the side at magnifications between 10× and 30×. Images with different focal planes were combined to generate an image with a high depth-of-focus.
